# Relaxation-expansion model for self-driven retinal morphogenesis: A hypothesis from the perspective of biosystems dynamics at the multi-cellular level

**DOI:** 10.1002/bies.201100070

**Published:** 2012-01

**Authors:** Mototsugu Eiraku, Taiji Adachi, Yoshiki Sasai

**Affiliations:** 1) Organogenesis and Neurogenesis Group, RIKEN Center for Developmental BiologyKobe, Japan; 2) Unit for Four-Dimensional Tissue Analysis, RIKEN Center for Developmental BiologyKobe, Japan; 3) Department of Biomechanics, Institute for Frontier Medical Sciences, Kyoto UniversityKyoto, Japan; 4) Computational Cell Biomechanics Team, VCAD System Research ProgramRIKEN, Wako, Japan

**Keywords:** ES cells, internal force, optic cup, retina, self-organization

## Abstract

The generation of complex organ structures such as the eye requires the intricate orchestration of multiple cellular interactions. In this paper, early retinal development is discussed with respect to the structure formation of the optic cup. Although recent studies have elucidated molecular mechanisms of retinal differentiation, little is known about how the unique shape of the optic cup is determined. A recent report has demonstrated that optic-cup morphogenesis spontaneously occurs in three-dimensional stem-cell culture without external forces, indicating a latent intrinsic order to generate the structure. Based on this self-organizing phenomenon, we introduce the “relaxation-expansion” model to mechanically interpret the tissue dynamics that enable the spontaneous invagination of the neural retina. This model involves three consecutive local rules (relaxation, apical constriction, and expansion), and its computer simulation recapitulates the optic-cup morphogenesis in silico.

## Introduction

**Figure 1:**
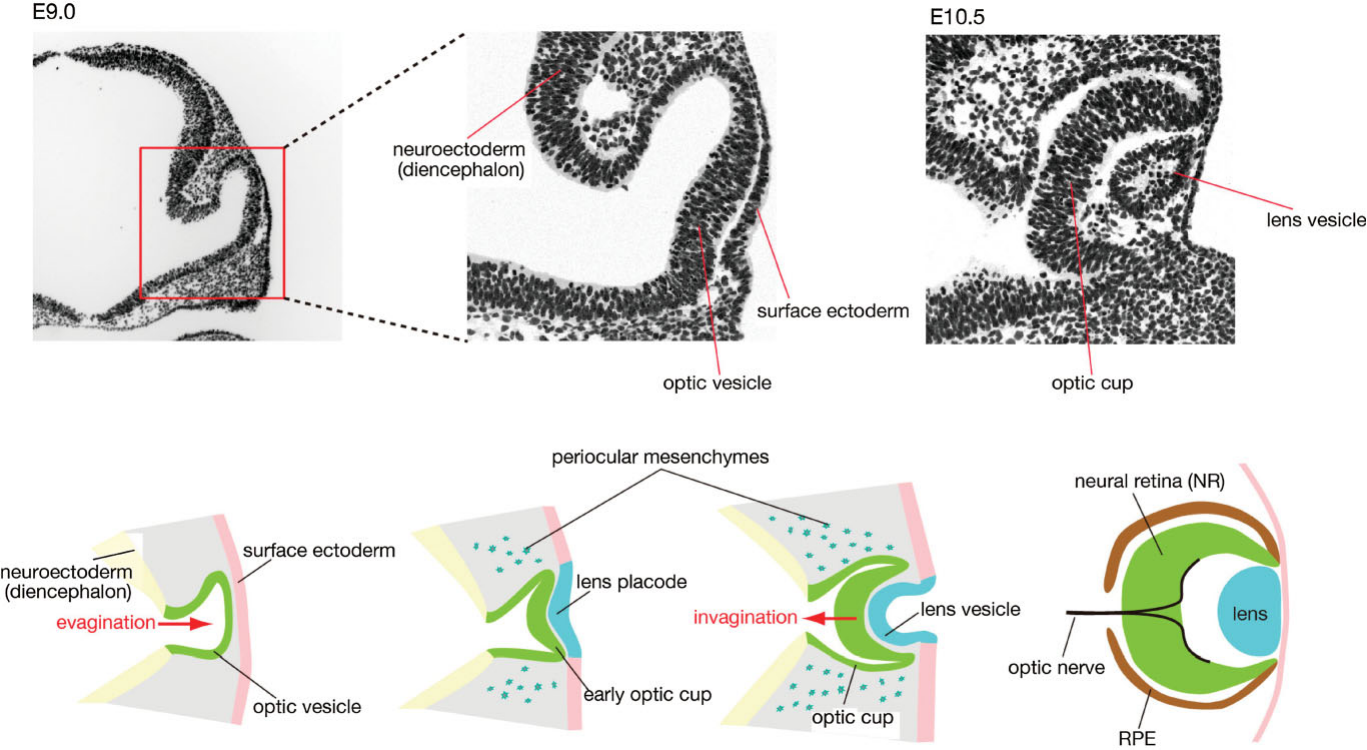
Early eye development. Schematic of early mammalian eye development. The optic vesicle forms as an epithelial sac evaginating laterally from the rostral diencephalic wall. The distal portion of the vesicle subsequently invaginates and becomes the neural retina (NR). By the evagination and invagination of the retinal epithelium, a double-walled cup structure (optic vesicle) is formed with the inner and outer walls being the NR and retinal pigment epithelium. At the same time, the surface ectoderm adjacent to the optic vesicle is fated to become a lens placode by inductive signals from the retinal progenitors, and then invaginates to form a lens vesicle. Upper panels, nuclear staining of cross-sectioned mouse embryonic heads.

During early embryogenesis, the retinal primordium arises from the rostral-lateral part of the diencephalon [1]. The monolayered retinal neuroepithelium evaginates laterally from the diencephalic wall to form an optic vesicle (Fig. 1). The distal portion of the vesicle, which is geometrically in contact with the surface ectoderm, is fated to become the neural retina (NR; sensorial tissue), while the proximal portion later differentiates into the retinal pigment epithelium (RPE; supporting tissue of the NR). The optic vesicle then invaginates at its distal portion to form a two-walled cup-like structure, the optic cup, with the NR and RPE being the inner and outer walls. At the same time as this infolding of the NR, the surface ectoderm adjacent to the retina also invaginates and develops into the lens vesicle, while the rest of the surface ectoderm near the lens becomes the corneal epithelium. During this process, neural crest-derived head mesenchymal cells accumulate in the periocular space [2]. Thus, early eye development involves several simultaneous events that take place within a tiny space in the embryonic head.

Eye formation has been a favorite topic in experimental embryology for many decades. For instance, lens induction from the surface ectoderm by the NR is a well-known subject of embryology giant Hans Spemann [3–5]. In addition to Spemann's ablation studies, Lewis elegantly presented the same principle in his ectopic graft study of optic vesicles in frogs [6]. On the other hand, the lens-inducing activity of the optic vesicle is not likely to drive all the events, and the responding ectoderm also needs to have a certain lens-forming competence. In Spemann's experiments using the frog neurula (*Bombinator*), lens formation was induced from a graft of head ectoderm, but not of trunk ectoderm, by the optic vesicle [5].

## Mechanism of optic-cup formation: A long-standing debate

Since the time of Spemann, the mechanism of eye-cup formation has been a matter of debate; many controversial models have been presented, in particular, regarding the necessity of non-retinal tissues, such as lens, surface ectoderm, or periocular mesenchyme. Importantly, when Spemann transplanted the trunk ectoderm in *Bombinator*, although no lens formed near the retina, the optic vesicle often still developed into the optic cup, suggesting that the optic cup could form without the concomitant generation of lens tissues in the adjacent space. This finding argued against the idea that the lens physically pushes the NR to bend inwards. However, these transplantation studies, including Spemann's lens-induction experiments, received substantial criticism at the time, and were challenged with contradicting results [3]. Spemann answered these criticisms by attributing such discrepancies to differences in conditions, including animal species. Some of these intriguing arguments can be read in his monograph of 1938 [5].

Since the classic embryology era, many embryologists have sought to understand the mechanism of coordinated eye-cup formation and its relation to neighboring tissues, including the lens, periocular mesenchymes, cornea and surface ectoderm, and diencephalon [7–9]. Some studies using chick and mouse embryos have suggested that the surface ectoderm and its derivates (e.g. lens) play roles in optic-cup formation [10–12], while others have indicated that the surface-ectoderm derivates, at least the lens, are not essential for NR invagination [13]. This argument is quite complex and requires careful considerations. For instance, the lens-specific depletion of Pax6 causes severe defects of both lens and optic-cup invaginations. However, this may not be simply the matter of direct mechanical interactions, since the lens-specific depletion of Pax6 also substantially affects the cellular properties of the retina in a non-cell-autonomous fashion [14]. Another intriguing study reported that contractile filopodia of lens epithelium tether lens and NR during lens invagination; however, loss of these filopodia increases the gap between the two epithelia, suggesting that pulling forces, rather than pushing forces (which could facilitate NR's invagination), are mediated by these connections [15].

Recent studies using zebrafish and medaka, whose transparent embryos provide a great advantage for live imaging, have revealed detailed information about the cell behaviors occurring during eye formation. The formation of the optic vesicle in teleosts is qualitatively different from that in mouse and chick embryos; for instance, instead of evaginating as in the mouse, fish retinal progenitor cells individually migrate out laterally from the neural tube and form a vesicle by local epithelialization [16]. The dependence of the NR invagination on surrounding tissues, however, has remained rather elusive in fish, as well [7].

## The optic cup can form in a self-organizing manner in vitro

The dynamic processes that form the optic cup occur in a small space and complex environment affected by many neighboring tissues. Therefore, it is inevitably difficult to determine whether the effect of an experimental manipulation in vivo (e.g. ablation) is direct or secondary. With this in mind, we sought to address this question by introducing an in vitro embryonic stem cell (ESC) culture system, which successfully reduced the complexity of this organogenetic process down to the internal control of epithelial morphogenesis.

Recent progress in ESC research has enabled the in vitro differentiation of neural and retinal progenitors as well as their derivatives [17–20]. When cultured in medium containing minimal extrinsic growth-factor signals, ESCs undergo selective neural differentiation in a manner dependent on the transcription factor Zfp521 [21]. These ESC-derived neural progenitors adopt the rostral forebrain fate (positive for Six3 [22, 23]) as a default fate, unless they are cultured in the presence of extrinsic caudalizing factors such as Wnts and Fgf [24–26].

**Figure 2:**
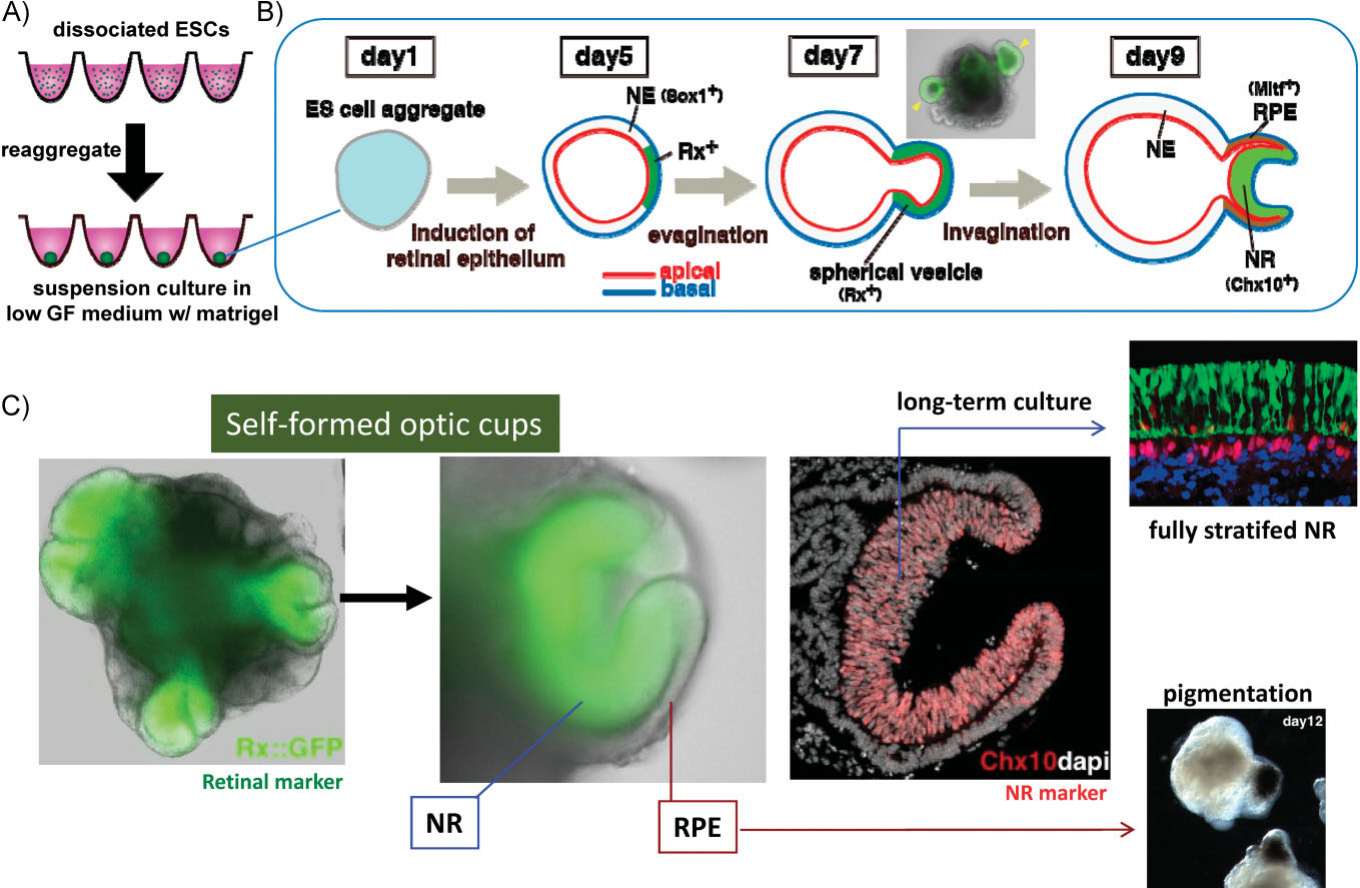
Self-organizing eye-cup formation from ESC aggregates. **A:** The SFEBq culture is started by the quick reaggregation of 3,000 mouse ES cells in a culture well. **B:** The SFEBq aggregate in retinal differentiation culture undergoes epithelialization, and parts of the epithelia start expressing the retinal marker Rx. The retinal epithelium, as in vivo, then evaginates to form a vesicle. The distal portion of the vesicle subsequently invaginates, resulting in formation of the optic-cup structure. **C:** Example of ESC-derived optic cups. Green, Rx::GFP for retinal marking. The aggregate has four optic cups formed on its surface, and each cup has an inner NR wall (Chx10^+^) and an outer RPE wall. In extended culture, the former becomes a fully stratified NR tissue, while the latter becomes pigmented like the RPE.

Floating aggregate culture of ESCs has the advantage of mimicking tissue formation in a three-dimensional (3D) context, and an efficient method for rostral forebrain differentiation is SFEBq culture (serum-free floating culture of embryoid body-like aggregates with quick reaggregation) [25, 27, 28]. To start this culture, a fixed number of dissociated ESCs (e.g. 3,000 cells for mouse ESCs) are reaggregated in each well of a 96-well plate that has a special surface coating for blocking cell-plate adhesion (Fig. 2A), and are cultured as a floating aggregate in serum-free medium. During the next few days, the ESCs first differentiate into epiblast cells (post-implantation pluripotent cells with epithelial properties) [21], and then the initially homogenous cell mass transforms into a hollow ball of single-layered epithelium [27, 28].

Efficient retinal differentiation occurs when SFEBq aggregates of mouse ESCs are cultured under low growth-factor conditions in a medium containing extracellular matrix proteins (matrigel) [28]. The addition of matrigel, whose major component is laminin, also appears to be advantageous for reinforcing the formation of basement membrane on the surface of the aggregate [29]. When cultured under these conditions, parts of the hollow neuroepithelial ball (usually, one to several patches per ball) start to express the retina marker Rx [30], on days 4-5 (Fig. 2B). On days 6-7, these Rx^+^ retinal epithelia evaginate to form vesicles. Then, during the next two days, the distal portion of each vesicle spontaneously invaginates, resulting in the formation of a double-walled cup structure reminiscent of the optic cup (Fig. 2C) [28]. In addition to having the right shape, the inner wall expresses NR markers such as Chx10, while the outer wall differentiates into RPE. Consistent with the in vivo topology, the inner side of the ESC-derived NR epithelium is apical (Fig. 2B, red lines). RPE differentiation is caused by soluble signals from the neighboring non-retinal neuroepithlium, which are likely to include Wnt factors 31–34.

The retinal progenitors in the NR undergo a characteristic interkinetic nuclear migration [28] as seen in vivo [35]. When ESC-derived neuroectoderm (NE) is isolated and cultured in retinal maturation medium, it generates a fully stratified NR structure with properly arranged layers containing six cell types in two weeks [27]. This self-forming nature of NR stratification may be consistent with previous studies showing that a reaggregate of retinal stem cells has an ability to generate a laminated sphere [36, 37].

Importantly, throughout the process of optic-cup formation in ESC culture, no lens or surface ectoderm is formed, and no substantial accumulation of mesenchyme is observed near the retina. Furthermore, an optic-cup structure can form when an optic vesicle is isolated from the SFEBq aggregate and cultured in Wnt-containing medium (which supports RPE differentiation) [28]. These observations strongly demonstrate that optic-cup formation in vitro occurs in a self-organizing fashion without physical influences from external structures such as the lens or surface ectoderm.

## What are the mechanics driving optic-cup morphogenesis in the epithelium?

**Figure 3:**
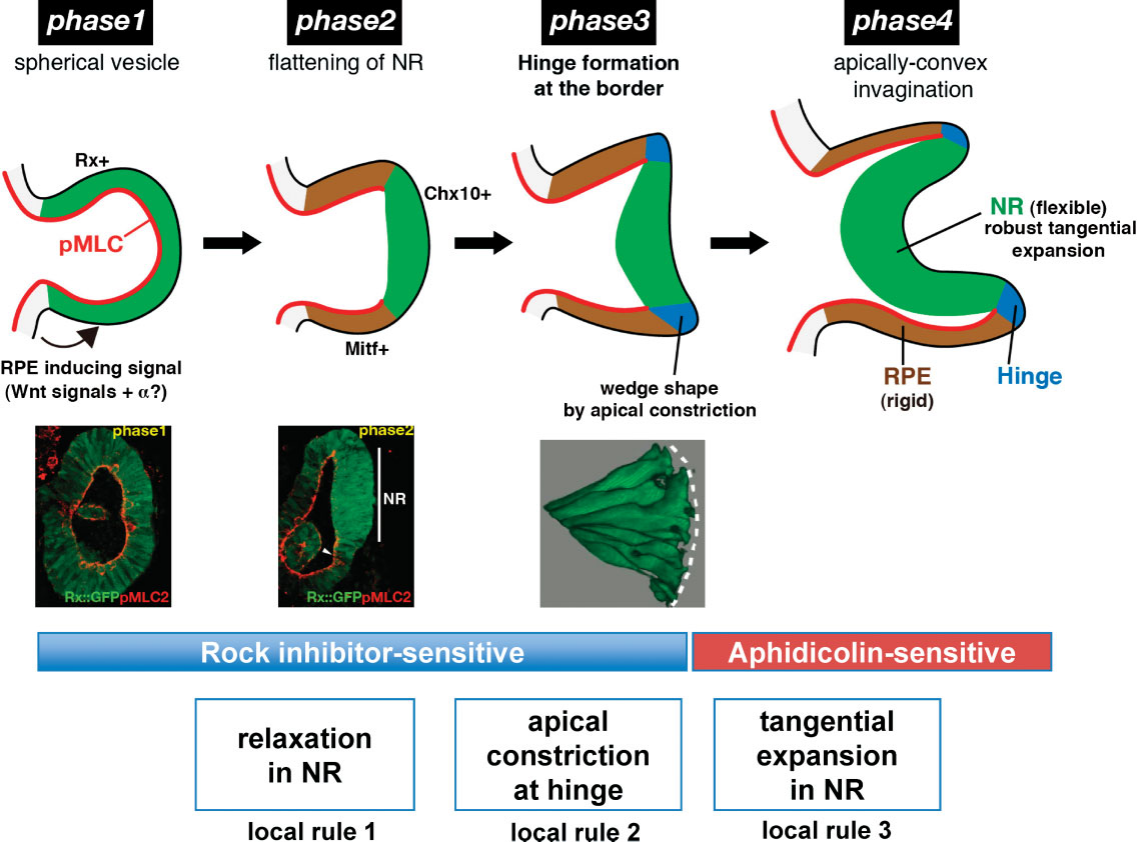
Four phases of invagination morphogenesis. Four phases of early retinal morphogenesis. A spherical vesicle (Phase 1) becomes patterned into presumptive RPE and NR along the proximal-distal axis, partly by inductive signals from non-retinal brain tissue, including canonical Wnt signals. The presumptive NR, located distally, down-regulates the level of pMLC and becomes morphologically flattened (Phase 2). Subsequently, the boundary epithelium between the NR and RPE domains (hinge) undergoes strong apical constriction and transforms into a wedge shape (Phase 3). Lastly, the NR substantially expands tangentially by rapid cell proliferation. The mechanically flexible NR surrounded by the rigid RPE shell bends inwards (invagination) in an apically convex fashion (Phase 4). Three local rules of tissue property changes are shown at the bottom.

If the optic vesicle is not forced to deform by external tissues, how does it transform into a cup structure by an internal mechanism? According to reproducible observations in detailed 3D live imaging using multi-photon optics, the invagination process of the NR in mouse ESC culture can be divided into four morphological phases (Fig. 3) [28]; similar stepwise changes in morphology are also seen in vivo [38]. On day 6 of culture, the evaginated Rx^+^ vesicle is hemispherical in shape and consists of uniform columnar epithelium (Phase 1). Around day 7, the presumptive NR at the distal portion of the vesicle becomes flattened (Phase 2). Subsequently, the joint region (hinge) between the NR and RPE domains undergoes apical constriction, and the apical angle of the hinge region becomes acute (Phase 3). During days 8-9, the NR epithelium progressively expands and invaginates as an apically convex structure (Phase 4). During these phases, the NR portion becomes a pseudo-stratified epithelium consisting of radial glia-like cells. The cells at the hinge region, which initially show a simple columnar morphology, transform into an apically narrow wedge shape upon the apical constriction at Phase 3.

At Phase 2, the initially homogenous-looking retinal epithelium first shows at least three region-specific differences. First, the curvature of the distal portion becomes flatter than that of the proximal portion. Second, the distal portion begins to express the NR marker Chx10 [39, 40], indicating that its differentiation is proceeding toward the NR fate. Third, the activation level of myosin, indicated by the accumulation of phosphorylated myosin light chain 2 (pMLC) [41, 42], is substantially decreased in the presumptive NR (immunostaining pictures in the middle row of Fig. 3). The low pMLC level in the NR portion continues all through Phases 2-4 (also observed in the developing mouse retina [28]).

In contrast, a strong accumulation of pMLC is observed homogeneously in the Phase 1 epithelium. This condition is considered to cause high tension in the actomyosin system within the optic vesicle. Rho kinase [Rho-dependent coiled-coil kinase (ROCK)] is known to regulate the pMLC levels for tension control within the epithelium in various cases [41, 42]. Accordingly, treatment with a ROCK inhibitor expands the size of the Phase 1 vesicle by relaxation. Importantly, the mechanical rigidity of the Phase 1 epithelium, as measured by an atomic force microscope (AFM)-based elasticity assay [28, 43, 44], is homogenously high, and this rigidity is substantially decreased by treatment with a ROCK or actomyosin inhibitor. Similarly, when the Phase 4 NR (low pMLC) and RPE (high pMLC) tissues are compared, the mechanical rigidity is much higher in the RPE than in the NR (Fig. 3), and inhibitor experiments showed that this difference is dependent on ROCK and actomyosin activity [28]. In addition, the apical constriction of the hinge epithelium, which results in the wedge-shaped morphology, does not appear in the presence of ROCK and myosin inhibitors.

These findings demonstrate a critical role for the spatiotemporal regulation of the ROCK/myosin-dependent microfilament system in the self-driven invagination morphogenesis. Indeed, when the culture is treated with the ROCK inhibitor Y27632 or the myosin inhibitor Blebbistatin before Phase 4, neither allows the invagination of the NR.

In contrast, the effect of Y27632 treatment is qualitatively different when it is started after the hinge is formed, and ROCK inhibition does not strongly block the progression of the NR invagination during Phase 4. Instead, treatment with the mitotic inhibitor aphidicolin strongly attenuates the invagination at this stage. During Phases 3-4, the entire NR and the distal RPE greatly expand in area (tangential expansion) in conjugation with rapid cell proliferation. Such robust proliferation can cause compression in the tangential direction within these epithelia. Consistent with this idea, microscopic tangential compression is observed in a cell ablation assay using a 3D-pinpointed multi-photon laser beam [28, 45]. The laser-ablated space in the Phase 4 NR and RPE is quickly filled in by lateral compression, whereas a gap created in Phase 1 epithelium remains open and even expands after ablation.

## The relaxation-expansion model for NR invagination

Based on these domain- and phase-specific changes in tissue properties, a hypothetical model was proposed to explain the self-driven morphogenesis occurring during optic-cup formation from the ESC-derived optic vesicle [28]. In this hypothesis, namely the “relaxation-expansion” model, three sequential local rules for changes in tissue properties (Fig. 3, bottom) are applied to drive the directed invagination movement of the NR epithelium during Phases 2-4.

As the initial condition, the evaginated optic vesicle at Phase 1 is considered to possess an even distribution of substantial tension within the epithelium regardless of its distal or proximal position. The pMLC accumulation is particularly high in the apical microfilament bundles; this conceivably contributes to the apically concave curvature of the Phase 1 vesicle by promoting a preferential narrowing (moderate) on the apical side.

As the first local rule for invagination, a substantial mechanical relaxation occurs in the distal retinal epithelium (presumptive NR) from Phase 2 and onwards because of the reduction in pMLC levels. This makes the invaginating NR epithelium more flexible than the RPE.

The second rule is based on the observed change in cell morphology; i.e. the strong apical constriction of the hinge epithelium at Phase 3. This causes the hinge cells to have an apically narrow wedge shape, and contributes to a slight apical (inward) bending of the NR epithelium at this stage.

The third rule is the tangential expansion of the NR during the late phases of eye-cup morphogenesis. During Phases 3-4, substantial cell proliferation in the NR and RPE causes their tangential expansion and generates some compression within the tissues. Whereas the expansion of the mechanically rigid RPE simply contributes to the distal extension of the outer wall of the cup, the area expansion of the flexible Phase 4 NR in a limited space is not a mechanically easy task, given that the less-yielding shell of RPE prevents the NR from undergoing its flat expansion. In this situation, the NR expansion causes strong buckling of the tissue, leading to invagination.

In the developing embryo, there are various tissues that undergo invagination. However, most of the invagination movements occur in an apically concave manner (e.g. neural-tube closure, lens invagination, and fly gastrulation) 46–49. Unlike these situations, the NR invagination proceeds with its apical side convex [28]. The low-pMLC context of the Phase 4 NR is probably an important factor enabling its unique, apically convex invagination, considering that strong tension in the apical microfilament bundle, if present, could resist the apical expansion. However, mechanical flexibility alone cannot account for the direction of the NR curve, because, in theory, the flexible tissue can bend in either direction, convex (invaginating) or concave (evaginating). This raises the important question of whether the apically convex curvature is an intrinsic NR property. However, this does not seem to be the case with mouse ESC-derived NR, because the NR tissue generated from an isolated Phase 1 vesicle bends in an apically concave manner but develops normally in other respects [28]. Therefore, in this current relaxation-expansion model, a small inward-bending bias is introduced as a result of the second rule, although whether the apical hinge constriction is an active or passive process still remains to be solved at present.

## Computer simulation as a proof-of-concept test

The relaxation-expansion model for NR invagination is consistent with the changes in regional tissue properties and can explain key mechanistic aspects of this unique tissue deformation with a few simple local rules applied sequentially. Then, can the three local rules of this model be sufficient to cause optic-cup morphogenesis in the ESC-derived retinal epithelium?

**Figure 4:**
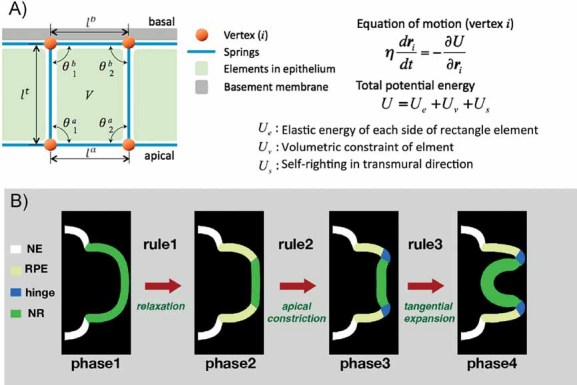
Computer simulation of the relaxation-expansion model. **A:** The spring-based vertex model used to simulate retinal epithelial morphogenesis according to the relaxation-expansion model. The retinal epithelium (optic vesicle) and neighboring non-retinal neuroepithelium at Phase 1 are considered. For this vesicular epithelium, 100 vertices are assigned per half dome on both the basal and apical surfaces, and neighboring vertices are connected by elastic springs in the tangential and transmural directions. The overdamped condition is assumed for the equation of motion, which determines the motion of each vertex to minimize the potentials for elastic energy, volumetric constraint, and element deformation in the transmural direction. *r_i_*, position vector of vertex *i*. The energy *U*_e_ is elastic strain energy of the springs expressed as 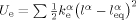, where *l*^α^ is the length of element side, 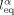 is its natural length at the stress-free state, and 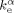 is the elastic constant for α = *a* (apical), *b* (basal), *t* (transmural). The energy *U*_v_ is for local volumetric change of the elements expressed as 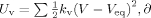 where *V* is the element volume, *V*_eq_ is the equilibrium element volume, and *k*_v_ is the volumetric elastic constant. The energy *U*_s_ is introduced to express the resistance to the distortion of the elements (the “self-righting” effect), as 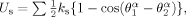 where 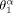 and 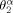 are the angles of element corners for α = *a* (apical), *b* (basal), and *k*_s_ is the elastic constant. **B:** Example of the 2D (pseudo 3D) simulation of epithelial deformation according to the three local rules.

To address this question, a computer simulation was performed as a proof-of-concept test, by virtually applying these local rules to a spring-based vertex model [28]. In this model, to reduce the geometric complexity, the optic cup structure is numerically demonstrated under the axisymmetric condition using a two-dimensional (2D) continuum model (Fig. 4A). The 2D model of epithelium consisting of multiple cells along the tangential direction is discretized into quadrilateral elements expressed by their vertex coordinates (100 vertices for the apical and basal surface, respectively, along the proximal-distal axis per half dome of the optic vesicle in a section). The vertices are connected by apical, basal, and transmural springs (Fig. 4A).

By solving the equation of motion under the overdamped condition (where the effect of acceleration is neglected): 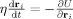 (η, virtual viscosity of the system; proposed by Honda et al. [50, 51]), the motions of vertices (d*r_i_*/d*t*, velocity vector) are determined to minimize the local potential energy of the system *U*, which is the sum of the potentials for elastic strain of the springs, volumetric change of the elements, and resistance to tangential distortion of the elements.

Initially (before optic-vesicle evagination; Phase 0), to form an apically concave curvature of the epithelium, the natural length of the apical spring is 14% shorter than that of the basal spring while their spring constants are the same (the transmural spring is 10 times longer and softer than the basal spring). At Phase 1, the apical spring in the retinal epithelium is further shortened by 10% (as compared to Phase 0) for fitting to a larger curvature of the optic vesicle. While the morphogenesis proceeds during Phases 1-4, these natural lengths and elastic constants are dynamically controlled in a domain- and phase-specific manner as described below. In addition, deformation of the epithelium generated by the hoop stress, which comes from the three-dimensionality of the vesicle, is taken into account by applying apparent force to each vertex in the radial direction.

To this virtual spring model of retinal epithelium, the three sequential changes in local rules are applied (Fig. 4B):

1. relaxation of the presumptive NR beginning at Phase 2, by gradually releasing the elastic strain energy stored in the tangential elements on the apical and basal surfaces (gradually relaxing their natural lengths); in addition, from the end of Phase 2 to Phase 3, the spring constants of the apical and basal springs in the NR region are gradually decreased down to 25% of Phase 0 in a linear fashion and kept low during invagination;
2. apical narrowing (constriction) in the hinge epithelium at Phase 3, by reducing the natural length of the apical spring there (down to 10% of Phase 0) and increasing the elastic constant (by 10-folds);
3. rapid tangential growth of the NR and the RPE (distal half) tissues during Phase 4, by linearly increasing the size (natural length and equilibrium volume) of the elements in the corresponding directions.

Notably, the sequential application of these simple procedures can mimic the tissue deformation of the NR during invagination morphogenesis (Fig. 4B); the NR spontaneously involutes inside the RPE shell as seen at Phase 4, following the NR flattening at Phase 2 and the hinge formation at Phase 3 [28]. Thus, this in silico model conceptually supports the feasibility of cup morphogenesis driven by internal rules in the absence of external forces. The three local rules are necessary for the simulation to generate the proper cup formation. For instance, when rules 1 and 2 are omitted, the NR evaginates, instead of invaginating, in the simulation. In culture, a perturbation of the microfilament system at Phases 2-3 also blocks the NR invagination [28].

## Remaining mechanistic questions and alternative interpretations

Whereas the current relaxation-expansion model is based upon the observations of three local changes in morphology, rigidity, and growth at the cell and tissue levels, it does not address how these local tissue behaviors are strictly controlled in a spontaneous fashion. For instance, it is unknown why the cells at the hinge region undergo strong apical constriction at Phase 3, or how the pMLC level decreases at Phase 2 specifically in the NR epithelium. To understand the domain- and phase-specific organization of the local rules, it will be important to combine the current tissue-level “relaxation-expansion” model with some underlying molecular-level models, in particular, for cytoskeleton regulations.

The essence of the relaxation-expansion model is that flexible NR epithelium, driven by its own expansion, buckles inside the shell of rigid (less yielding) RPE. In this case, the direction of bucking is given by a minor bias for inward curving in NR at the end of Phase 3. Based on the morphological change in 3D live imaging, the current relaxation-expansion model postulates that the strong apical constriction of the hinge promotes this small bias for apical-directed NR infolding. However, the model still has a relative arbitrariness in the explanation of the curving direction, since more than one mechanism could be still considered for the cause. For instance, in theory, a similar inward bias may also be generated by biased apical expansion and/or basal constriction of NR epithelium 52–54, while some resistance of NR's basement membrane to tangential expansion also favors apically convex curving. So far, exact contributions of these alternative mechanisms to the in vitro self-formation remains elusive. For instance, although basal constriction of NR epithelium has been shown to contribute to the formation of fish optic cups [54], an obvious accumulation of actomyosin or pMLC2 is not observed on the basal side in the ESC-derived NR, while ROCK inhibitor applied during Phase 4 does not have strong effects on its invagination [28]. On the other hand, biased apical expansion may be caused, in theory, by preferential apical accumulation of cell bodies or higher mechanical compliance on the apical side, both of which are intriguing topics for future investigation.

Regarding the infolding dynamics of NR, another intriguing observation was reported in fish optic-cup formation; a portion of nasal NR epithelia undergoes compaction, while the temporal portion of the outer epithelium ingresses over the hinge into the inner cup to contribute to the formation of temporal NR [55]. Such a flow of cell migration and compaction was not found during the optic-cup formation from ESCs [28]. The issue of the mechanistic conservation and diversity across species should certainly await further in vivo studies.

## Possible improvements in quantitative and mathematical aspects of the model

Regarding quantitative and mathematical aspects, further efforts for improvements may be necessary to overcome several limitations of the current model. First, the current model is mostly based on qualitative or semi-quantitative data, and the same is true for its computer simulation. It could be enough for the proof of concept to support the theoretical feasibility of spontaneous cup deformation without external forces. However, for high-precision simulation, it will be important to include quantitative parameters such as real elasticity constants in the apical, basal, and transmural directions, as well as the exact numbers and sizes of cells. Second, the current model does not take the plastic nature of NR (found in ref. [28]) into consideration. In the ESC-derived optic cup, the RPE and hinge regions are elastic and evert by residual stress when the cup is excised by cutting at the proximal RPE. In contrast, NR epithelium is plastic and does not change its shape upon excision, suggesting that residual stress (expected from microscopic compression by proliferation) is relatively rapidly released within the tissue when seen at the macroscopic level. The mechanism for it (e.g. microscopic cell rearrangement) and its mathematical expression are important questions to be addressed. Another weakness of the current simulation is its assumption of the axisymmetric condition for the optic cup to reduce the structural complexity into the 2D (or pseudo-3D) context; 3D mechanical modeling should be more suitable for understanding more real and complex mechanics. Lastly, the model does not involve mechanical properties of the basement membrane or its effects on NR invagination.

In addition, to mathematically treat these complex aspects of deformation in a strict manner, it may be also necessary to consider several intricate issues as exemplified below:

- Local stress-free reference configurations and statistical indeterminacy in tissue with their spatial inhomogeneity.
- Nonlinearities in tissue mechanical properties and geometrical changes due to large deformation.
- History-dependent control of tissue viscosity properties.
- Mechanical feedback to the morphogenesis (for instance, local growth rates and/or actomyosin activity might be a nonlinear function of stress and strain).
- Precise addition and deletion of material points (e.g. by cell division and apoptosis).
- Positive involvement of noise to overcome local energy barriers.

Because of this complex mixture of nonlinearities (including bifurcations), establishing the evolution equations for tissue volume, geometries, properties, and stress-free configurations is a demanding matter. However, we believe that this difficult task is worth the effort in the long run at least for the optic-cup morphogenesis, particularly because the simple self-organization system from ES cells, which includes a relatively limited number of elements as compared to the in vivo eye, has becomes available for in vitro analysis.

## Conclusions and perspectives

In this paper, we have discussed a model for the unexpected self-organizing nature of optic-cup formation. The self-organization mechanism is sufficient for optic-cup development in vitro, and the high reproducibility of the tissue pattern and structure [56] strongly argues against it being an in vitro artifact.

Nonetheless, we do not wish to claim that the self-organizing mechanism is the only one that functions for in vivo retinogenesis. The high order of robustness necessary for the in vivo generation of a precise organ such as the eye can presumably be achieved by linking multiple principles in combination. For instance, it is likely that optic-cup invagination is regulated more strictly in space and time if it has the double assurance of optic-cup self-organization and the spatial constraints of lens invagination [15]. This is an intriguing topic in future investigation.

## Abbreviations

AFM: atomic force microscope
2D: two-dimensional
3D: three-dimensional
ESC: embryonic stem cell
NE: neuroectoderm
NR: neural retina
pMLC: phosphorylated myosin light chain 2
ROCK: Rho-dependent coiled-coil kinase
RPE: retinal pigment epithelium

## References

[1] Graw J (2010). Eye Development. Curr Top Dev Biol.

[2] Fuhrmann S, Levine EM, Reh TA (2000). Extraocular mesenchyme patterns the optic vesicle during early eye development in the embryonic chick. Development.

[3] Hamburger V (1988). The Heritage of Experimental Embryology.

[4] Spemann H (1901). Über Korrelationen in der Entwicklung des Auges. Ver Anat Gesellsch.

[5] Spemann H (1938). Embryonic Development and Induction.

[6] Lewis WH (1907). Experimental studies on the development of the eye in Amphibia I. On the origin of the lens in *Rana palustrius*. Am J Anat.

[7] Martinez-Morales JR, Wittbrodt J (2009). Shaping the vertebrate eye. Curr Opin Genet Dev.

[8] Fuhrmann S (2010). Eye morphogenesis and patterning of the optic vesicle. Curr Top Dev Biol.

[9] Adler R, Canto-Soler MV (2007). Molecular mechanisms of optic vesicle development: complexities, ambiguities and controversies. Dev Biol.

[10] Lopashov GV, Medawar J (1963). Developmental Mechanisms of Vertebrate Eye Rudiments.

[11] West-Mays JA, Zhang J, Nottoli T, Hagopian-Donaldson S (1999). AP-2alpha transcription factor is required for early morphogenesis of the lens vesicle. Dev Biol.

[12] Smith AN, Miller LA, Radice G, Ashery-Padan R (2009). Stage-dependent modes of Pax6-Sox2 epistasis regulate lens development and eye morphogenesis. Development.

[13] Hyer J, Kuhlman J, Afif E, Mikawa T (2003). Optic cup morphogenesis requires pre-lens ectoderm but not lens differentiation. Dev Biol.

[14] Ashiery-Padan R, Marquardt T, Zhou X, Gruss P (2000). Pax6 activity in the lens primordium is required for lend formation and for correct placement of a single retina in the eye. Genes Dev.

[15] Chauhan BK, Disanza A, Choi SY, Faber SC (2009). Cdc42- and IRSp53-dependent contractile filopodia tether presumptive lens and retina to coordinate epithelial invagination. Development.

[16] Rembold M, Loosli F, Adams RJ, Wittbrodt J (2006). Individual cell migration serves as the driving force for optic vesicle evagination. Science.

[17] Ikeda H, Osakada F, Watanabe K, Mizuseki K (2005). Generation of Rx+/Pax6+ neural retinal precursors from embryonic stem cells. Proc Natl Acad Sci USA.

[18] Lamba DA, Karl MO, Ware CB, Reh TA (2006). Efficient generation of retinal progenitor cells from human embryonic stem cells. Proc Natl Acad Sci USA.

[19] Idelson M, Alper R, Obolensky A, Ben-Shushan E (2009). Directed differentiation of human embryonic stem cells into functional retinal pigment epithelium cells. Cell Stem Cell.

[20] Osakada F, Ikeda H, Mandai M, Wataya T (2008). Toward, eneration of rod- and cone-photoreceptors from human and monkey embryonic stem cells under defined culture conditions. Nat Biotechnol.

[21] Kamiya D, Banno S, Sasai N, Watanabe K (2011). Intrinsic transition of ES cell differentiation into neural progenitors. Nature.

[22] Lagutin OV, Zhu CC, Kobayashi D, Topczewski J (2003). Six3 repression of Wnt signaling in the anterior neuroectoderm is essential for vertebrate forebrain development. Genes Dev.

[23] Lagutin O, Zhu CC, Furuta Y, Rowitch DH (2001). Six3 promotes the formation of ectopic optic vesicle-like structures in mouse embryos. Dev Dyn.

[24] Watanabe K, Kamiya D, Nishiyama A, Katayama T (2005). Directed differentiation of telencephalic precursors from embryonic stem cells. Nat Neurosci.

[25] Wataya T, Ando S, Muguruma K, Ikeda H (2008). Minimization of exogenous signals in ES cell culture induces rostral hypothalamic differentiation. Proc Natl Acad Sci USA.

[26] Muguruma K, Nishiyama A, Ono Y, Miyawaki H (2010). Ontogeny-recapitulating generation and tissue integration of ES cell-derived cerebellar Purkinje cells. Nat Neurosci.

[27] Eiraku M, Watanabe K, Matsuo-Takasaki M, Kawada M (2008). Self-organized formation of polarized cortical tissues from ESCs and its active manipulation by extrinsic signals. Cell Stem Cell.

[28] Eiraku E, Takata N, Ishibashi H, Kawada M (2011). Self-organizing optic-cup morphogenesis in three-dimensional culture. Nature.

[29] Fujiwara H, Hayashi Y, Sanzen N, Kobayashi R (2007). Regulation of mesodermal differentiation of mouse embryonic stem cells by basement membranes. J Biol Chem.

[30] Bailey TJ, El-Hodiri H, Zhang L, Shah R (2004). Regulation of vertebrate eye development by Rx genes. Int J Dev Biol.

[31] Martinez-Morales JR, Rodrigo I, Bovolenta P (2004). Eye development: a view from the retina pigmented epithelium. BioEssays.

[32] Westenskow P, Piccolo S, Fuhrmann S (2009). Beta-catenin controls differentiation of the retinal pigment epithelium in the mouse optic cup by regulating Mitf and Otx2 expression. Development.

[33] Liu W, Lagutin O, Swindell E, Jamrich M (2010). Neuroretina specification in mouse embryos requires Six3-mediated suppression of Wnt8b in the anterior neural plate. J Clin Invest.

[34] Fujimura N, Taketo MM, Mori M, Korinek V (2009). Spatial and temporal regulation of Wnt/beta-catenin signaling is essential for development of the retinal pigment epithelium. Dev Biol.

[35] Norden C, Young S, Link BA, Harris WA (2009). Actomyosin is the main driver of interkinetic nuclear migration in the retina. Cell.

[36] Nakagawa S, Takada S, Takada R, Takeichi M (2003). Identification of the laminar-inducing factor: Wnt-signal from the anterior rim induces correct laminar formation of the neural retina in vitro. Dev Biol.

[37] Layer PG, Robitzki A, Rothermel A, Willbold E (2002). Of layers and spheres: the reaggregate approach in tissue engineering. Trends Neurosci.

[38] Hilfter SR, Yang J-JW (1980). Anat Rec.

[39] Livesey FJ, Cepko CL (2001). Vertebrate neural cell-fate determination: lessons from the retina. Nat Rev Neurosci.

[40] Cayouette M, Poggi L, Harris WA (2006). Lineage in the vertebrate retina. Trends Neurosci.

[41] Amano M, Ito M, Kimura K, Fukata Y (1996). Phosphorylation and activation of myosin by Rho-associated kinase (Rho-kinase). J Biol Chem.

[42] Nishimura T, Takeichi M (2008). Shroom3-mediated recruitment of Rho kinases to the apical cell junctions regulates epithelial and neuroepithelial planar remodeling. Development.

[43] Rico F, Roca-Cusachs P, Gavara N, Farré R (2005). Probing mechanical properties of living cells by atomic force microscopy with blunted pyramidal cantilever tips. Phys Rev E: Stat Nonlinear Soft Matter Phys.

[44] Krieg M, Arboleda-Estudillo Y, Puech PH, Käfer J (2008). Tensile forces govern germ-layer organization in zebrafish. Nat Cell Biol.

[45] Mascaro AL, Sacconi L, Pavone FS (2010). Multi-photon nanosurgery in live brain. Front Neuroenerg.

[46] Sawyer JM, Harrell JR, Shemer G, Sullivan-Brown J (2010). Apical constriction: a cell shape change that can drive morphogenesis. Dev Biol.

[47] Kinoshita N, Sasai N, Misaki K, Yonemura S (2008). Apical accumulation of Rho in the neural plate is important for neural plate cell shape change and neural tube formation. Mol Biol Cell.

[48] Haigo SL, Hildebrand JD, Harland RM, Wallingford JB (2003). Shroom induces apical constriction and is required for hingepoint formation during neural tube closure. Curr Biol.

[49] Hildebrand JD (2005). Shroom regulates epithelial cell shape via the apical positioning of an actomyosin network. J Cell Sci.

[50] Honda H, Tanemura M, Nagai T (2004). A three-dimensional vertex dynamics cell model of space-filling polyhedra simulating cell behavior in a cell aggregate. J Theor Biol.

[51] Nagai T, Honda H (2009). Computer simulation of wound closure in epithelial tissues: cell-basal-lamina adhesion. Phys Rev E: Stat Nonlinear Soft Matter Phys.

[52] He L, Wang X, Tang JL, Montell DJ (2010). Tissue elongation requires oscillating contractions of a basal actomyosin network. Nat Cell Biol.

[53] Gutzman JH, Graeden EG, Lowery LA, Holley HS (2008). Formation of the zebrafish midbrain-hindbrain boundary constriction requires laminin-dependent basal constriction. Mech Dev.

[54] Martinez-Morales JR, Rembold M, Greger K, Simpson JC (2009). ojoplano-mediated basal constriction is essential for optic cup morphogenesis. Development.

[55] Picker A, Cavodeassi F, Machate A, Bernauer S (2009). Dynamic coupling of pattern formation and morphogenesis in the developing vertebrate retina. PLoS Biol.

[56] Ali RR, Sowden JC (2011). DIY eye. Nature.

